# Walsh’s Physicians’ Handy Ledger

**Published:** 1880-01

**Authors:** 


### BIBLIOGRAPHICAL.

WALSH'S PHYSICIANS' HANDY LEDGER, a companion to physicians, combined Call Book and Tablet. Published by Ralph Walsh, M.D., Washiugton, D.C., 326 Northwest street. Mailed prepaid, upon receipt of $3.00.

This is a neatly bound volume of three hundred pages, and is the most convenient arrangement for book-keeping that could be furnished a doctor. Each day's work may be entered in such a manner that at any time an estimate can be made with little or no trouble. We would not be without it.

